# To Evaluate Serum Eosinophil Cationic Protein and Total IgE Concomitantly May Predict the Persistence of Wheezing in Young Children

**DOI:** 10.5402/2012/168379

**Published:** 2012-12-24

**Authors:** Esengül Keleş, Hamza Yazgan, Arzu Gebeşçe

**Affiliations:** Department of Pediatric Health and Diseases, Sema Application and Research Hospital, Fatih University, Istanbul, Turkey

## Abstract

*Background*. We investigated the predictive value and the relative risk of the evaluation of serum eosinophilic cationic protein (sECP) and total IgE levels concomitantly in relation to the persistence of wheezing in young children. *Methods*. The study was conducted prospectively between January 2007 and December 2010. A hundred and eight children, aged between three months and four years, with three or more episodes of wheezing, were studied to evaluate the role of eosinophil inflammation and its relation to persistence of wheezing two years later. *Results*. A statistically significant difference in terms of total IgE and sECP values was observed between the groups (*P* < 0.05). When measurement of IgE and sECP was assessed concomitantly, the sensitivity was found to be 92.68%, the negative predictive value was found to be 71.43%, accuracy rate was found to be 84.26%, and the relative risk was found to be 3.06 in group 1. *Conclusions*. In this study, we aimed to emphasize the importance of the assessment of sECP and total IgE concomitantly, as being two noninvasive and easily applicable tests, useful in predicting persistent wheezing in early childhood.

## 1. Introduction

 Wheezing is a musical sound frequently heard during expiration. It is heard in the form of a prolonged whistle and occurs mostly together with diseases that constrict the lower respiratory tract and rarely the upper respiratory tract. Wheezing is a frequent complaint among patients seeking admission to hospital. It often occurs in early childhood, although it may be observed in people of all ages. Chronic or recurrent wheezing may be caused by several etiological factors. It is a controversial topic as to whether or not children who wheeze in their early childhood will also develop asthma in the future. 

 Asthma generally starts during the first years of life. However, young wheezing children are a heterogeneous group. About 60% of early wheezers suffer transitory disease and become asymptomatic when they reach 5-6 years of age, with only 40% continuing with asthma at this age. Identifying infants who will go on to develop persistent wheezing and determining whether inhaled anti-inflammatory drugs, or other treatments, can block the processes leading to chronic asthma are important challenges in the prevention of this common disease. Conventional modalities remain incapable of diagnosing and following up people with asthma. Respiratory function tests and prick tests, which are commonly used to establish clinical diagnoses, exhibit some difficulties when applied to young children. Therefore, assessments directed to eosinophils, which are the essential cells of inflammation, and their products have gained in importance. The quantitative measurement of ECP in serum, which is a granular eosinophil product, has been reported to be a specific indicator of inflammation of the respiratory tract [1, 2]. The levels of sECP, which is one of the leukocytic granular proteins, have been found to be high during the course of many active inflammatory diseases. sECP, which is mostly produced by eosinophils, is toxic to the neurons and to some of the epithelial cell walls. Unlike total IgE, there is a good correlation between sECP concentration and clinical findings. sECP levels do not directly correlate with the peripheral eosinophil count. However, sECP levels are closely related to the activity of the eosinophils. Numerous studies report this as a valuable parameter in displaying inflammation of the respiratory tract and eosinophilic activation in children with asthma [3]. 

 Our study was designed to evaluate the importance of eosinophilic inflammation in wheezing children in the first years of life and to identify factors at the onset of the disease, including measurement of sECP, as a marker of eosinophilic inflammation, and total IgE, concomitantly, as possible predictors for the persistence of wheezing at age of 5 years.

## 2. Patients and Methods

 Study participants were outpatients between three months and four years of age who came to the Private Fatih University Sema Application and Research Hospital between January 2007 and December 2010, having had three or more episodes of wheezing. Patients with a clinical history suggestive of other respiratory problems, or heart disease, those with neonatal pathology, and those who had taken systemic or inhaled corticosteroids or cromoglycate in the previous three months were excluded. A hundred and eight patients (60 boys and 48 girls) were enrolled in the study. While forming the study and control groups the patients were not evaluated on the basis of presence of allergic rhinitis, atopic dermatitis, or a history of maternal asthma. Blood samples were drawn during wheezing attacks.

 Two years later, at a second clinical evaluation, the children were separated into two groups. We classified children as persistent wheezers if they had had at least one wheezing episode over the past six months (autumn and winter) or if they had cough or dyspnea with exercise (group 1, persistent wheezers). The second group was children who had been asymptomatic during the past six months (group 2, transient wheezers). 

 Measurements of 24 mcg\lt and higher were accepted as high for sECP. Simultaneously, a total IgE measurement of 15 IU\mL and higher was accepted as high for zero to 12 months, while values of 60 IU\mL and higher were accepted as high for 13 to 60 months. Eosinophilic cationic protein (ECP) levels in the serum were measured, using the automated chemiluminescent immunoassay system (Immulite 2000 analyzer) immunologic test analyzer, with the solid phase, two-site, “chemiluminescent immunometric” method. Serum total IgE levels were measured with the electrochemiluminescence immunoassay immunological test (ECLIA), using the sandwich principle and cobas e601 immunology test analyzer. 

## 3. Statistical Analysis 

 The Number Cruncher Statistical System (NCSS) 2007, the Power Analysis and Sample Size (PASS), 2008 Statistical Software (Utah, USA) programs were used for the statistical analysis. Significance was assessed at a level of *P* < 0.05. 

## 4. Results 

 In terms of the patients' gender distribution, no statistically significant difference was observed between group 1, which consisted of 46 male and 36 female patients, and group 2, which consisted of 14 male and 12 female patients (*P* < 0.05). In terms of age distribution, while the mean age of group 1 was found to be 2.65 ± 1.86 years and the mean age of group 2 was found to be 2.07 ± 2.04 years, no statistically significant difference was observed between the two groups (*P* > 0.05). The mean sECP values were found to be 45.9 in group 1 and 18.6 in group 2. The IgE value was found to be 97.1 in group 1 and 16.9 in group 2 (*P* < 0.01) (Table 1). 

 In relation to the measurement of ECP, 24 mcg/lt and higher was taken as high, and a statistically significant difference was observed between the rate of 68.3% in group 1 and of 15.4% in group 2 (*P* < 0.01). When considering IgE levels of 15 IU/mL and higher to be high for zero to 12 months and 60 IU/mL and higher to be high for 13 to 60 months, a significant difference was detected between the rate of 54.9% for total IgE in group 1 and the rate of 30.8% in group 2 (*P* < 0.05).

 While the probability of one of the measurements of IgE and/or sECP being high in group 1 was found to be 92.7% and 42.3% in group 2, a statistically significant difference was found between the two groups (*P* < 0.01). The evaluation of total IgE and sECP concomitantly revealed a sensitivity rate of 92.68%, a negative predictive value of 71.48%, an accuracy rate of 84.26%, with a highest relative risk of 3.06. The sECP measurement displayed a highest specificity rate of 84.62%, and a positive predictive value of 93.33%. At followup after two years, 73.5% of the patients in group 1 and 18.5% of the patients in group 2 showed persistence in wheezing. A statistically significant difference was found between the two groups in terms of the rate of persistent wheezing (*P* < 0.01) (Table 2). 

## 5. Discussion

 The degree to which eosinophil-mediated inflammation is involved in young children with asthma and the extent to which such inflammation will determine the prognosis remain unknown. Serum ECP is a mediator of the allergic inflammation, released by the eosinophils and activated by the mediation of IgE [4]. Numerous studies report sECP as a helpful parameter in displaying inflammation of the respiratory tract and eosinophilic activation in children with asthma [5]. 

 In a prospective study, 33 children with wheezing but without atopy, 15 children with upper respiratory tract infection but without atopy, and 18 healthy children were compared in terms of sECP values. The sECP values of the group with wheezing were found to be statistically significantly higher in comparison to the other two groups. Observations that lasted for one year revealed the beginning of the development of asthma in the group with wheezing. In conclusion, the sECP measurements in children with wheezing were considered to have predictive value for the development of asthma [6]. 

 In a prospective study conducted on 92 patients in Finland, sECP was found to be specifically high in acute attacks of wheezing [7]. 

 In 2010, in Italy, the sECP levels of 441 cases of respiratory tract disease were investigated retrospectively and compared to 33 healthy infants. The sECP levels were found to be significantly higher in cases with asthma. However, the sECP levels were not found to be high in respiratory tract diseases other than asthma. The sECP sensitivity was found to be 70%, and specificity in predicting asthma was found to be 74% [8]. In our study, sensitivity was found to be 68.29%, while specificity was found to be 84.62%. 

 Numerous other articles, apart from those mentioned above, have also reported that the scope of sECP is a helpful parameter in displaying the inflammation of the respiratory tract and eosinophilic activation in children with asthma. However, the number of articles that have presented an opposite opinion has increased in recent years: for example, in a study conducted in 2007, an account was given of 47 cases of hospital admission for wheezy breathing (group 1); at least 3 wheezing attacks had occurred in 43 cases (group 2); and hospital admission, due to a cause other than respiratory system diseases, had occurred in 43 cases (the control group). In the groups (1 and 2) with wheezing, sECP levels were found to be statistically significantly higher. The groups with wheezing were investigated, and no statistically significant difference was found in sECP between the group with a first attack and the group with the recurrent attacks [9]. 

 The sECP levels and eosinophil counts of a total of 25 cases having a first bronchiolitis attack, aged one to 17 months, were evaluated. At the end of the followup, which lasted for three years, eight of the 25 had developed asthma. However, 15 of this group did not develop asthma. No significant difference was observed between the sECP levels and eosinophil counts of the two groups [10]. Khadadah et al. [11] evaluated the total IgE and sECP levels, as well as the peripheral eosinophilic count, concomitantly in a study involving 101 patients with asthma.

 In our study, the concomitant evaluation of the total IgE and sECP revealed a sensitivity rate of 92.68%, a negative predictive value of 71.48%, and an accuracy rate of 84.26%. The highest relative risk was 3.06, due to persistent wheezing at the end of the two-year followup period, which gave a rate of 73.5%. 

 It is now suggested that early treatment of bronchial inflammation, soon after the onset of the disease, may arrest its progress [12]. Most asthmatics start developing symptoms at age of 2-3 years, but 60% of children wheezing at this age will be asymptomatic when they are 6 years old, so it would be of interest to know which children have a transitory disease and which have early childhood asthma. The latter group would probably benefit from anti-inflammatory drug treatment. 

 Our findings suggest that eosinophilic inflammation is present from the onset of the disease in the group of wheezing young children who will continue with wheezing episodes at age 5-6 years. Determination of serum eosinophilic cationic protein and total IgE, concomitantly, was not evaluated on the basis of presence of allergic rhinitis, atopic dermatitis, or history of maternal asthma. This may help to determine which children will continue with asthma and could therefore benefit from a more aggressive approach to treatment and which children merit a more conservative approach, as their disease will probably be transitory. Studies with more patients and a longer follow-up period are needed to confirm these results. 

## Figures and Tables

**Table 1:** General baseline characteristics of the study groups.

	Study group	Control group	*P* value
Male gender	46 (56.1%)	14 (53.8%)	*P* > 0.05
Female gender	36 (43.9%)	12 (46.2%)	*P* > 0.05
Mean age (years)	2.65 ± 1.86	2.07 ± 2.04	*P* > 0.05
Total IgE	97.1 IU*∖*mL	16.9 IU*∖*mL	*P* < 0.01
sECP	45.9 mcg*∖*lt	18.6 mcg*∖*lt	*P* < 0.01

**Table 2:** Diagnosis screening results being displayed concomitantly.

	Sensitivity	Specificity	Positive predictive	Negative predictive	Accuracy	Relative risk
Total IgE	54.88	69.23	84.91	32.73	58.33	1.26
h sECP	68.29	84.62	93.33	45.83	72.22	1.72
IgE and ECP	92.68	57.69	87.36	71.43	84.26	3.06
